# Informing a sinusitis clinical trial protocol: A focus group study with clinicians and staff

**DOI:** 10.1017/cts.2024.663

**Published:** 2024-11-27

**Authors:** Ruey-Ying Liu, Charles Fencil, Tom Whitfield, Daniel Merenstein, Bruce Barrett, David Rabago, Alex H. Krist, Sebastian T. Tong, Aleksandra E. Zgierska, Derjung M. Tarn

**Affiliations:** 1 Department of Sociology, National Chengchi University, Taipei, Taiwan; 2 Department of Family Medicine and Population Health, Virginia Commonwealth University, Richmond, VA, USA; 3 MedStar Health Research Institute, Washington, DC, USA; 4 Department of Family Medicine, Georgetown University Medical Center, Washington, DC, USA; 5 Department of Family Medicine and Community Health, University of Wisconsin–Madison, Madison, WI, USA; 6 Department of Family and Community Medicine, The Pennsylvania State University, Hershey, PA, USA; 7 Department of Family Medicine, University of Washington, Seattle, WA, USA; 8 Department of Family Medicine, David Geffen School of Medicine, University of California–Los Angeles, Los Angeles, CA, USA

**Keywords:** pragmatic clinical trial, stakeholder engagement, qualitative method, focus group, acute rhinosinusitis

## Abstract

This study investigates practicing clinician and staff perspectives on potential protocol modifications for the “Nasal Irrigation, Oral Antibiotics, and Subgroup Targeting for Effective Management of Acute Sinusitis” (NOSES) study, a pragmatic randomized controlled trial aiming at improving acute rhinosinusitis management. Focus groups with clinicians and staff at the pretrial stage recommended expanding participant age inclusion criteria, incorporating patients with COVID-19, and shortening the supportive care phase. Participants also discussed patient engagement and recruitment strategies. These practical insights contribute to optimizing the NOSES trial design and underscore the value of qualitative inquiries and healthcare stakeholder engagement in informing clinical trial design.

## Introduction

Randomized controlled trials (RCTs) have long been recognized as the gold standard for gauging the effectiveness and efficacy of interventions, [1] yet they often encounter hurdles in real-world clinical scenarios. Only 50% of RCTs meet their recruitment targets, and 25% do so in a timely manner [2,3]. Factors contributing to low recruitment rates include inadequate communication about trial design, overestimation of eligible participants, and perceived participant burden [4,5]. To address these factors, researchers are increasingly turning to pragmatic trials conducted in real-world settings, assessing the broader effectiveness of treatment with a more generalizable patient population [6].

The “Nasal Irrigation, Oral Antibiotics, and Subgroup Targeting for Effective Management of Acute Sinusitis” (NOSES) study is a pragmatic RCT evaluating treatment options for acute rhinosinusitis (ARS), a leading cause of antibiotic usage in primary care. Antibiotics are prescribed in over 70% of outpatient ARS visits and incur $11 billion in direct annual costs [7–12]. Previous investigations on ARS treatments were limited by explanatory designs, modest sample sizes, and a lack of diverse perspectives to inform methodology [10,13,14]. Key issues such as determining the appropriate eligibility criteria for trial participants and managing logistical support in treatment administration remain challenging.

NOSES is a 6-site, 4-arm randomized placebo-controlled double-blind pragmatic trial designed to compare the effect of antibiotics alone, antibiotics with intranasal corticosteroids, intranasal corticosteroids plus placebo, or placebo, on ARS outcomes. Participants also receive supplies for saline nasal irrigation (SNI) devices. The proposed protocol was developed by academic partners at six primary care academic centers (Georgetown University, Pennsylvania State University, University of California-Los Angeles, University of Washington, University of Wisconsin-Madison, and Virginia Commonwealth University), leveraging their expertise in clinical trials and ARS evidence. NOSES is funded by the Patient-Centered Outcomes Research Institute (PCORI)’s Pragmatic Clinical Trials program.

The present study aims to inform decisions regarding protocol refinement by conducting focus groups with nonacademic clinicians and staff who address ARS as part of their routine clinical practice. This approach aligns with the growing recognition of clinician engagement at the pretrial stage of RCTs, enabling researchers to identify potential recruitment issues, provide a more nuanced understanding of clinics and communities, and facilitate the transferability of findings [15,16]. Additionally, this approach is consistent with PCORI’s recommendation to include healthcare stakeholder engagement in study design and implementation [17].

## Method

We conducted focus groups with practicing physicians, advanced practice providers (APPs), and clinic staff (practice managers, front office staff, and medical assistants) who were not part of the NOSES research team. Focus groups were convened about 6 months prior to commencing recruitment for NOSES. Each of the six participating sites referred 2–4 participants for each focus group. The Georgetown University’s Institutional Review Board approved this study (#00005886).

The focus groups were conducted online via Zoom from April to May 2023. They lasted approximately 90 minutes and were video-recorded. Focus group interviews were moderated by an external patient engagement expert and co-moderated by one of the study investigators (DM). The moderators, with input from the study team, developed two semi-structured interview guides with open-ended questions: one tailored for physicians and advanced practice practitioners (APPs), focusing on questions about medical aspects of the trial design, and the other for staff, focusing on patient recruitment and staff engagement. The focus groups commenced with a 15-minute NOSES overview covering study aims and originally proposed trial design, followed by discussions using the appropriate focus group guide.

Table 1 outlines the guiding questions presented to the focus groups, which queried participants about four aspects of the trial design: (1) Should the eligibility criterion of a maximum age of 65 years (as per existing clinical practice guidelines [18]) be increased? (2) Should the trial include patients diagnosed with COVID-19 (not covered by existing practice guidelines [18])? (3) Should the duration of the supportive care phase, that is, the period from enrollment to treatment initiation, be shortened from 10 days (as per existing practice guidelines [18]) to 7 days? (4) What type of SNI wash device, a neti pot versus a squeeze bottle, is optimal for trial participants? In addition, we sought focus group participants’ opinions on promoting patient recruitment and staff engagement. Participants responded to these open-ended questions and interacted with each other to explore and clarify their opinions. Each participant received $200 for their involvement.

**Table 1.** Key semi-structured focus group questions

Introductory question	What questions do you have about the study activities?
Maximum age for participant recruitment	What do you think about the inclusion criteria related to age?
Inclusion of patients with COVID-19	What is your advice about including patients with COVID-19?*
Duration of supportive care phase for sinusitis	What do you think about asking patients to receive supportive care for 10 days?**
Saline nasal irrigation wash device	We are going to give out free nasal wash materials. Should we offer squeeze bottles, neti pots, or both?**
Practice workflow	What might cause a problem or disrupt the flow of work in your clinic setting?
Recruitment strategy	What is your advice on how to make the whole recruitment of patients and participants as successful as possible in your clinic setting?
Staff training and engagement	What do you think would be a good way for our project team to engage staff and clinicians in your clinic?
Final thought	Any final thoughts on how to make this study work best in your clinic setting?

* For physicians only.

** For physicians and advanced practice practitioners only.

Focus group recordings were transcribed using Microsoft Stream and verified by two investigators (RL and TW). A sociologist with expertise in qualitative research (RL) and a health research analyst (TW) independently reviewed all transcripts and used inductive content analysis, a bottom-up exploratory approach, to categorize participant responses into themes [19]. The two investigators compared their analyses and came to a consensus on the themes, which were then reviewed collectively by the study team. ATLAS.ti 23 (ATLAS.ti GmbH) was used to code the qualitative data.

## Results

We conducted three focus groups: one with physicians (n = 10), one with APPs (n = 5), and one with clinic staff (n = 8). Table 2 depicts focus group feedback on the trial design, and Table 3 illustrates themes related to participant recruitment.

**Table 2.**
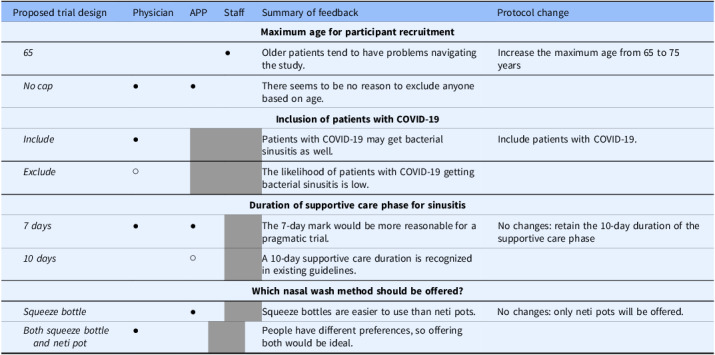
Focus group feedback regarding trial design, summary, and resultant project changes, by type of participant*

APP = advanced practice practitioner.

* A solid dot (●) indicates that all or a majority of participants expressed support for the proposed study design; a hollow dot (○) indicates that a minority of focus group participants supported the proposed study design; and gray shading indicates that the topic was not raised during the focus group.

**Table 3.** Participant suggestions for optimizing patient recruitment, with example quotes

Major topic related to optimizing patient recruitment	Participant comments	Example quotes
Practice workflows	Schedule is likely to be disrupted.	“I’m pretty concerned about the enrollment process …You have to think hard about where and when you’re going to do the enrollment, and if you do it before and slow things down, that’s not going to work. People are going to get really grumpy because then they’re late and then they’ve got the next patient lined up.” (Participant 5, Physician)
	Limited space availability is a major issue for smaller practices.	“There are days where we have enough flexibility that we could have let a room go for an hour and not have it be a problem. And then there are other times where that would bog down the whole system.” (Participant 6, Staff)
	Incorporating the recruitment process in telehealth would be more difficult, but most practices have scaled back telehealth.	“I’m doing very little telehealth at this point, and when I am, it’s not really for URI-type things…We were seeing all of our URI stuff telehealth initially but now as long as they’ve had a negative COVID test within 24 hours for bringing them into the clinic. So I think that that would help with recruitment.” (Participant 1, APP)
Recruitment strategies	Distributing flyers with QR codes	“Having some sort of flyer with a QR code that people could scan while they’re in the room and just bring some information right up on their phone.” (Participant 3, Physician)
	Providing cheat sheets to clinicians/staff	“If I were involved recruiting patients, I would want like a little card or something that I can be referencing or handling to the patient with like 3 sentences about this.” (Participant 7, Physician)
	Utilizing the patient portal or mass email invitations	“We’ve had some success with our quality measures in the office getting those numbers up by using the patient portal with their recommendations… maybe like a mass e-mail, like if you develop these please reach out, we have this research study that you may qualify.” (Participant 2, Staff)
	Tailoring recruiting strategies to different sites	“With the patient population we work with, which is largely just poor, a lot of them do not have good phones or reliable internet access at home. Sending them home with something to sign online probably would not be an effective recruitment method.” (Participant 2, APP)
Staff training and engagement	Lunch-and-learn events	“Food is the love language at my clinic, so if there is an opportunity to bring in a lunch and do like a lunch and learn.” (Participant 6, Staff) “For our clinic setting like prior to it starting having a lunch-and-learn type because we rarely get together…so like a meal and kind of a lunch-and-learn and then obviously the flyers and once the person’s here like everyone else said, kind of the daily introduction and just that continued communication with the staff is important.” (Participant 1, APP)
	Incentive programs (for individuals or the team)	“If you have like a certain amount of people enrolled in your office and based on how many people then they get, not that you’d come up with prizes per se, but certain things, whether it be monetary compensation or two lunches or whatever and then they have goals and then incentivize them that way.” (Participant 2, Physician) “I like the idea of involving the whole office as opposed to one specific nurse… something more for the whole team and it gives you something to talk about like how many did we get this week, how many more till we get to the next level or something, and that keeps it the forefront of patient of people’s minds.” (Participant 9, Physician)
	Connection between research staff and the clinical team	“Engagement from the get-go, from the initial person coming in, the assistant coming in should really take time to engage with the front desk staff and the amazing kind of say why they’re there. Otherwise, they’re kind of just a person kind of lurking around that they signed somebody up for something.” (Participant 4, APP)

### Trial design

#### Maximum age for participant recruitment

Most physicians and APPs recommended against using age as an exclusion criterion and advocated for including patients over 65 years of age in NOSES. However, a minority of participants shared concerns related to including older patients. A few staff advocated for excluding patients over 65, expressing concerns that older patients might have difficulty following instructions and tend to seek antibiotics more often.

#### Inclusion of participants diagnosed with COVID-19

Only the physician group discussed the inclusion of patients testing positive for COVID-19. Physicians overwhelmingly agreed to include patients with COVID-19, citing no theoretical ground for excluding them. Only one participant argued against including patients with COVID-19 because “the likelihood that they have an acute bacterial sinusitis on top of COVID is pretty low.” (Participant 1, Physician)

#### Duration of supportive care phase

Physicians and APPs had extensive discussions about the desired duration of the supportive care phase. While acknowledging the existing guideline recommendation of a 10-day waiting period before starting antibiotics, participants generally noted that seven days would be more reasonable for a pragmatic trial. One physician captured the view shared by many:

I try to push people towards ten, but usually by seven, they’re pushing pretty hard that they think they need something else and you’re going to get a lot of people getting upset. I think if you stick with seven, more people will stay with the study. (Participant 7, Physician)

#### Nasal irrigation device

Physicians and APPs lacked consensus about which SNI wash device (neti pot or squeeze bottle) should be offered to participants, as many noted that this is a matter of personal preference. Several APPs supported offering squeeze bottles, while physicians generally advocated for allowing patients to choose between the two.

### Trial participant recruitment

#### Practice workflows during recruitment

Many focus group participants expressed concerns regarding potential disruptions to practice workflows. Participants discussed strategies to optimize recruitment and reduce disruptions to clinician schedules, but solutions varied across practice sites. Some participants suggested stationing research assistants in a dedicated room and having clinic staff refer patients to them, whereas those from smaller practices with limited space noted that such procedures “would bog down the whole system” (Participant 6, Staff).

Participants also discussed using telehealth for recruitment. Most participants indicated that their practices have fewer telehealth visits than in previous years and that their patients are currently encouraged to have in-person visits for upper respiratory infections. Some clinics had a clinician dedicated to telehealth; for these clinics, developing a recruitment process was considered relatively straightforward.

#### Staff engagement

Participants across all focus groups noted that the best way to engage clinicians and staff is by bringing food. Providing food in a group setting is “very team building” (Participant 4, Physician), “the love language in my clinic” (Participant 6, Staff) and “goes a really long way” (Participant 3, APP).

Lunch-and-learn events were widely praised, but some physicians pointed out that a one-time session may not yield sustained engagement. Developing an incentive program with prizes, either individual- or team-based, may help keep the study “at the forefront of people’s minds” (Participant 9, Physician).

Participants also emphasized the importance of building relationships between the research team and clinic staff from the outset of recruitment. Such connections would raise staff’s awareness of the study and thus help prevent investigators from missing potential trial participants.

#### Recruitment strategies

Participants suggested several strategies for boosting patient recruitment, including distributing flyers with QR codes, providing information sheets for staff and clinicians, and utilizing patient portals or mass email invitations. They emphasized the importance of tailoring recruitment strategies to each site’s patient population since patients’ backgrounds and expectations are likely to influence recruitment strategies. The following quotes exemplify views about trial recruitment for two distinct patient populations:

With the patient population we work with, which is largely just poor, a lot of them don’t have good phones or reliable internet access at home. (Participant 2, APP)

We have lots of, I mean tons of researchers coming in and out all the time from our clinic… [Patients are] used to talking to people, talking to students, talking to researchers. It’s just part of coming to receive care at a teaching hospital. (Participant 3, APP)

## Discussion

Focus group interviews with physicians, APPs, and clinic staff provided valuable input for NOSES, a pragmatic RCT of ARS treatments, leading to protocol changes to include patients with COVID-19 and to increase the maximum age for participant recruitment from 65 to 75 years. The suggestion to shorten the supportive care phase before starting treatments from 10 to 7 days, while clinically pragmatic, was not implemented – in collaboration with the funder, the study team opted not to make the protocol change since it was aligned with the existing practice guideline [18]. In addition, the investigators decided to offer only neti pots and not squeeze bottles for SNI supportive care. Recommendations that were not adopted nonetheless offer valuable considerations for future pragmatic trials involving ARS treatment.

Focus group participants also shared insights for optimizing patient recruitment and staff engagement, drawing from their understanding of local needs and resources in the clinical setting. These suggestions were adopted to engage clinicians and staff in the recruitment process and to facilitate trial recruitment. Moreover, involving clinicians and staff at the participating recruitment sites in this focus group study may increase their awareness of the trial and bolster their engagement in it.

This study confirms the value of qualitative methods for informing pragmatic changes to trial design, as noted in previous RCTs, [15,16] and highlights the importance of engaging clinicians and staff. Although the use of qualitative methods in designing RCTs has increased considerably over the past few decades, many qualitative studies fail to demonstrate whether or how their findings lead to changes in trial design and implementation [15]. Often, findings are presented as lessons learned for future trials but are not articulated in ways that other researchers could readily implement [16]. This report explicitly illustrates clinician and staff feedback that informed the decision-making process of a subsequent trial. This inclusive approach can be applied to clinical trials in other areas.

This study has several limitations. We identified a consensus of themes across the focus groups but were unable to evaluate theoretical saturation (when no themes arise from the data) due to the limited number of focus groups conducted, that is, only one each with physicians, APP, and staff. Moreover, the sample may not be fully representative, as we did not collect demographic information from participants, and some sites may have contributed more participants to the focus groups than others.

In conclusion, our study underscores the valuable role of healthcare stakeholder engagement in RCT design and implementation. The involvement of trial stakeholders in the pretrial phase holds promise for offering pragmatic suggestions for trial design, developing effective recruitment strategies, and enhancing research engagement. Stakeholder insights warrant careful consideration by both research teams and funding agencies alike. PCORI has supported a transformational change in research, exemplified by this study, that is pragmatic and adaptive by partnering with healthcare stakeholders who will be involved in clinical trials. Other funders should consider similar approaches.
